# Genome-wide DNA methylation analysis for diabetic nephropathy in type 1 diabetes mellitus

**DOI:** 10.1186/1755-8794-3-33

**Published:** 2010-08-05

**Authors:** Christopher G Bell, Andrew E Teschendorff, Vardhman K Rakyan, Alexander P Maxwell, Stephan Beck, David A Savage

**Affiliations:** 1 Medical Genomics, UCL Cancer Institute, University College London, London, UK; 2 Institute of Cell and Molecular Science, Barts and The London School of Medicine and Dentistry, Queen Mary University of London, London, UK; 3 Nephrology Research Group, Centre for Public Health, Queen's University Belfast, Belfast, Northern Ireland, UK

## Abstract

**Background:**

Diabetic nephropathy is a serious complication of diabetes mellitus and is associated with considerable morbidity and high mortality. There is increasing evidence to suggest that dysregulation of the epigenome is involved in diabetic nephropathy. We assessed whether epigenetic modification of DNA methylation is associated with diabetic nephropathy in a case-control study of 192 Irish patients with type 1 diabetes mellitus (T1D). Cases had T1D and nephropathy whereas controls had T1D but no evidence of renal disease.

**Methods:**

We performed DNA methylation profiling in bisulphite converted DNA from cases and controls using the recently developed Illumina Infinium^® ^HumanMethylation27 BeadChip, that enables the direct investigation of 27,578 individual cytosines at CpG loci throughout the genome, which are focused on the promoter regions of 14,495 genes.

**Results:**

Singular Value Decomposition (SVD) analysis indicated that significant components of DNA methylation variation correlated with patient age, time to onset of diabetic nephropathy, and sex. Adjusting for confounding factors using multivariate Cox-regression analyses, and with a false discovery rate (FDR) of 0.05, we observed 19 CpG sites that demonstrated correlations with time to development of diabetic nephropathy. Of note, this included one CpG site located 18 bp upstream of the transcription start site of *UNC13B*, a gene in which the first intronic SNP rs13293564 has recently been reported to be associated with diabetic nephropathy.

**Conclusion:**

This high throughput platform was able to successfully interrogate the methylation state of individual cytosines and identified 19 prospective CpG sites associated with risk of diabetic nephropathy. These differences in DNA methylation are worthy of further follow-up in replication studies using larger cohorts of diabetic patients with and without nephropathy.

## Background

Diabetic nephropathy is a serious microvascular complication of diabetes and has become the most common cause of end-stage renal disease (ESRD) in many national ESRD registries [1,2]. Approximately one third of diabetic individuals will develop clinically apparent nephropathy characterised by persistent proteinuria, hypertension and eventual progressive decline in glomerular filtration rate [3,4]. Whilst chronic hyperglycaemia is integral to the pathogenesis of diabetic nephropathy there is also strong evidence for a genetic susceptibility to this common complication of diabetes [5-7]. In genetically susceptible individuals, prolonged hyperglycaemia leads to chronic metabolic and haemodynamic changes [8,9] whose effects, including those driven by transforming growth factor beta 1 (TGFβ1), promote structural abnormalities in the kidney such as glomerular basement membrane thickening, podocyte injury, and mesangial matrix expansion, with the later development of irreversible glomerular sclerosis and tubulointerstitial fibrosis [10].

Genetic studies have found evidence for linkage to nephropathy at a number of chromosome loci including 2q, 3q22 and 19q [11,12]. While no consistently replicated genetic associations have been identified, various candidate gene associations in diabetic nephropathy in type 1 diabetes (T1D) have been proposed, such as the SNP rs13293564 G/T substitution in intron 1 of *UNC13B *[13], and *SOD1 *SNPs [14]. An association with the *ELMO1 *gene and diabetic nephropathy was initially identified in patients with type 2 diabetes mellitus [15] and this association has now also been reported for diabetic nephropathy in T1D [16]. A recent genome-wide association study identified *FRMD3 *and *CARS *gene variants as possible genetic susceptibility factors for T1D diabetic nephropathy [17].

Epigenetic mechanisms allow alteration of genome function without mutating the underlying sequence. They involve the interacting actions of DNA methylation (the addition of a methyl group to the 5th carbon position of cytosine), histone modifications and non-coding RNAs [18]. A number of indirect lines of evidence point to the involvement of epigenetic changes in diabetic nephropathy. Murine models of disease progression displaying temporal variation in gene expression have indicated these supra-sequence devices may be involved in the pathogenesis [19]. Gene expression changes reflect dynamic alterations in gene transcription and also messenger RNA stability, which may be influenced by the epigenetic modification of the genome in response to chronic hyperglycaemic stress. Altered DNA methylation has been additionally implicated in vascular disease [20,21]. Furthermore, characteristics observed in diabetic nephropathy such as hyperhomocysteinaemia, dyslipidaemia, inflammation and oxidative stress can promote aberrant DNA methylation [22-24].

DNA methylation in mammalian species, except for pluripotent stem cells [25,26], appears to occur almost exclusively at consecutive CpG dinucleotides, of which there are approximately 28 million located within the human genome [27]. The majority of cytosines in these dinucleotides remain methylated (~80% in *Homo sapiens*) [27], which contrasts with CpG islands (CGIs) in which cytosines are generally unmethylated. CGIs are regions that exceed genome average levels of CpG density and co-localise with 72% of gene promoters [28], notably in those of highly expressed genes.

In order to assess possible changes in CpG methylation in a genome-wide manner we utilised the high throughput Illumina Infinium^® ^Human Methylation27 BeadChip DNA methylation array to investigate association with nephropathy in a case-control study of 192 T1D patients. These probes interrogate the methylation state of 27,578 individual CpGs located predominately in CGIs within proximal promoter regions, between 1.5 kb upstream and 1 kb downstream of the transcription start sites of 14,475 consensus coding sequence genes throughout the genome. Furthermore 110 miRNA promoters are targeted with 254 CpG loci probes [29]. This platform enables the high-throughput array based investigation of individual CpGs at a magnitude higher level than the previous array based Golden-Gate assay [30].

The development, as well as the progression, of diabetic nephropathy has been linked to the same inflammatory cell processes that are implicated in the progression of T1D itself, such as the activated T cells involved in the destruction of islet β cells [7]. Thus changes in the expression of cytokines and other inflammatory markers from distinct blood cell types make the investigation of methylation changes in DNA derived from blood a plausible option for examining epigenetic alterations in this disease. DNA methylation changes in peripheral blood have additionally been shown to indicate or be surrogate markers of active disease processes [31].

This study has investigated the possibility to identify DNA methylation biomarkers in peripheral blood cell-derived DNA that may be associated with the pathogenesis of T1D nephropathy. The study was designed to detect associations between the disease and differences in DNA methylation but not their functional relationship. Undertaken in a case control design, those with and without nephropathy were matched by age of onset and duration of diabetes.

## Methods

### Participants

Cases (n = 96) and controls (n = 96) were recruited from nephrology and diabetic clinics in Belfast and Dublin (Table 1). Genomic DNA was extracted from whole blood samples from cases and controls using a salting out method. All participants were white, with parents and grandparents born in Northern Ireland or the Republic of Ireland. Both cases and controls were diagnosed with T1D before the age of 31 years and required insulin from time of diagnosis. The definition of a case was based on development of persistent proteinuria (> 0.5 g protein/24 h) at least 10 years after diagnosis of diabetes, hypertension (BP > 135/85 mmHg or treatment with antihypertensive agents) and presence of diabetic retinopathy. In contrast, a control was defined as a patient with T1D duration of at least 15 years with urinary albumin excretion in the normal range and not receiving antihypertensive treatment. Patients with microalbuminuria were excluded from both groups. Cases and controls were matched for confounders that may affect DNA methylation namely age, gender and duration of diabetes within five year windows. Study approval for each recruitment site was obtained from respective research ethics committees (Queens University Belfast and Mater Misericordiae Hospital, Dublin) and written informed consent was obtained from all participants.

**Table 1:** Subjects

	Controls	Cases
Number	96	96

Age	43.2	42.8

Sex (M/F)	47/49	48/48

Age at Diagnosis	16.3	16.2

Duration T1D	27.8	27.5

Subjects: Number, Average Age, Sex proportion (Male/Female), Duration of T1D

### Bisulphite conversion of DNA

Bisulphite conversion of 1 ug genomic DNA was performed using the EZ-96 DNA Methylation-Gold™ Kit (^©^Zymo Research Corp., USA) in two 96 well plates according to the manufacturer's instructions. Cases and controls were randomly located between the two plates. Effective bisulphite conversion was checked by successful PCR amplification with a pair of primers specific for converted DNA and unsuccessful amplification with a pair of primers for unconverted DNA, of 24 random samples across both plates (details available on request from authors).

### Illumina Infinium methylation assay

DNA samples from cases and controls were interrogated utilising the Illumina Infinium^® ^Human Methylation27 BeadChip. This platform detects the methylation status of 27,578 CpG sites by sequencing-based genotyping of bisulphite treated DNA. The chromosomal distribution and other statistics relevant to these CpG sites are provided in Additional File 1: Supplementary Figure S1. This chemical modification of DNA converts only unmethylated cytosines to uracils, thereafter allowing for highly multiplexed genotyping with single site resolution. Bisulphite converted and unconverted (i.e. methylated) sites are simultaneously evaluated by hybridisation of DNA to site-specific probes attached to beads, one for unmethylated and the other for methylated sites, followed by allele specific base extension that includes a fluorescent label. Two different labels are used, and fluorescent signals are specific for either the T (unmethylated) or C (methylated) alleles. Methylation scores represented as β values are generated for each site using BeadStudio 3.2 software (^©^Illumina, Inc. 2003-2008) and are computed based on the ratio of methylated to methylated plus unmethylated signal outputs. Thus the β values range from 0 (unmethylated) to 1 (fully methylated) on a continuous scale.

This platform for quantitative methylation, essentially an adaptation of the highly successful Illumina Infinium I Whole Genome Genotype SNP genotyping assay used extensively for GWAS [32,33], is extremely accurate and powerful. Reproducibility estimates are extremely high with an average r^2 ^correlation for β-values of 0.992 for 24 technical replicates [29]. Due to the high density of probes up to 12 separate samples can be assayed on a single BeadChip, thus allowing for high-throughput processing.

The Illumina Infinium methylation assay was performed according to the manufacturer's instructions, and this along with the technical validation of the assay is detailed in Bibikova *et al*. [29]. Bisulphite-converted DNA was denatured, amplified, fragmented and subsequently hybridised, with use of a specific hybridisation buffer, to the chip arrays (Sentrix positions A-L). Cases and controls were arranged to randomly approximate a 50:50 proportion on each chip. Primer extension was performed, then staining and coating, followed by imaging with the Illumina BeadArray reader. Internal quality controls included assessment of staining, hybridisation, target removal, extension, bisulphite conversion efficiency, dye specificity and additionally negative controls.

### Statistical analysis

Initial array results were visualised using Illumina^® ^BeadStudio 3.2 (^© ^Illumina Inc 2003-2008). All computations and statistical analyses were performed using the R package (R 2.8.1) (http://www.r-project.org) [34] and Bioconductor [35].

#### Quality control and data normalisation

Quality control and normalisation of methylation data was undertaken using the analysis pipeline reported elsewhere [31]. Briefly, samples were monitored for coverage (fraction of CpGs with detectable intensity values above background) and bisulphite conversion efficiency (BSCE) using the controls provided on the Illumina Beadchip. Of the 192 samples, one sample had failed BSCE and an additional four samples had relatively low coverage and BSCE. These samples were removed, leaving a total of 187 samples with approximately 81% global coverage (22,486 CpG sites). Intra-array normalisation was performed by the Illumina BeadStudio 3.2. software. The resulting β-valued data matrix was normalised further using a quantile-normalisation strategy, designed to reduce unwanted inter-array variation. The normalised and raw data are available from GEO (Gene Expression Omnibus, NCBI) under the accession number GSE20067.

#### Singular Value Decomposition and Significance of Singular Values

**Figure 1:**
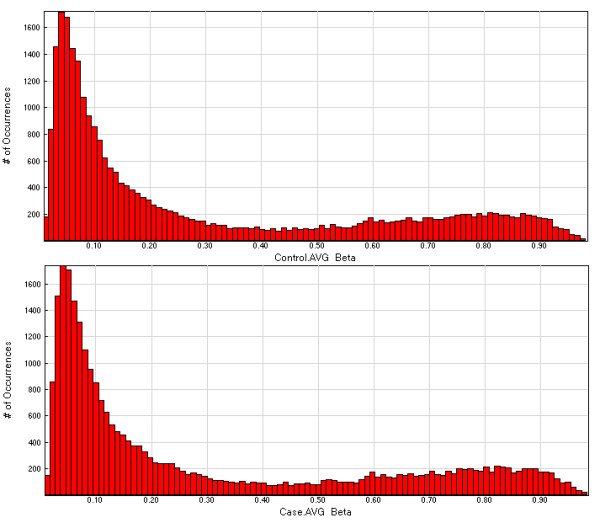
**Average β values**. Figure 1a (top): Controls, Figure 1b (bottom): Cases - Average β values across array.

**Figure 2:**
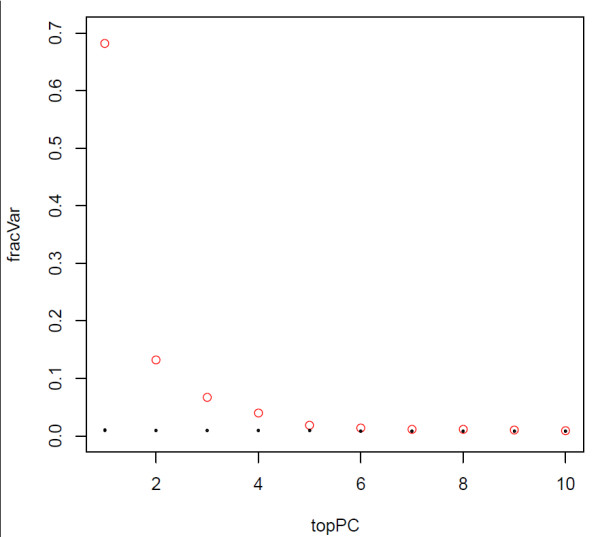
**Fraction of variation associated with principal components**. Fraction of variation associated with principal components after adjustment for the top component which captures the inherent bi-modality of the methylation distribution. Red denotes the observed values, black denotes those obtained by randomly scrambling up the data matrix. Each black dot represents the value of 10 distinct randomisations, which all yield the same value as singular values represent measures of global variation which themselves are invariant under a global randomisation of the data.

**Figure 3:**
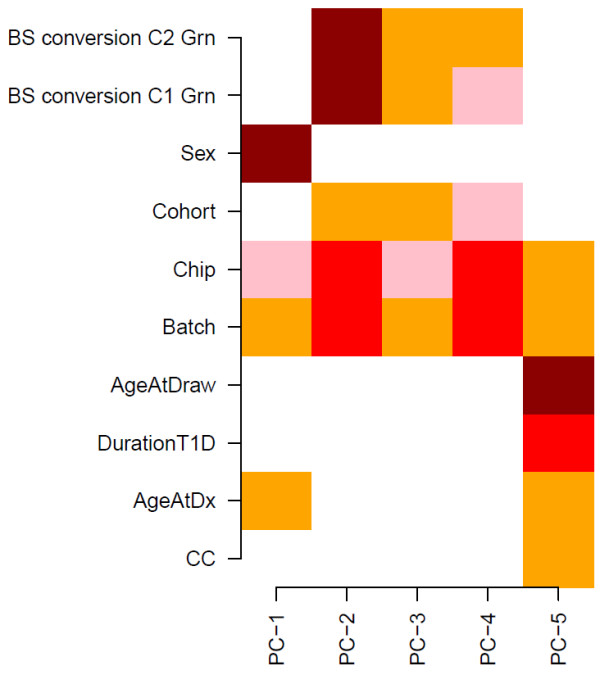
**Correlation of principle components to phenotype/experimental factors**. Correlation of principal components (top component removed as it did not correlate with any phenotype) to phenotypes of interest and experimental factors: BS conversion (bisulphite conversion, C1(green), C2(blue)), Sex, Cohort, Chip, Batch, Age at Draw (Age when sample taken), Duration T1D, Age at Diagnosis, CC (time to onset of NP) Colour key: dark red = p value < 10^-10^, red = p value < 10^-5^, orange = p value < 10^-2^, pink = p value < 0.05, white = p value > 0.05.

Singular Value Decomposition (SVD) provides a linear representation of the data in terms of a relatively small number of components, which capture the most salient patterns of variation [36]. We verified that the first component of a SVD analysis on the β-valued data matrix captures over 90% of the variation in the data, representing the inherent bi-modality of the methylation value distribution (see Figures 1a and 1b). Thus, to correctly evaluate the statistical significance of the remaining components of variation, the normalised data matrix needs to be adjusted by removing this background variation, by subtracting out the top principal component. This is essentially equivalent to normalising the data matrix by the mean profile. SVD was subsequently reapplied to the normalised adjusted data and the spectrum of singular values compared to the null distribution obtained by considering random matrices [37,38]. Specifically, the normalised adjusted data was randomised by permuting the CpGs, using a distinct permutation for each sample. Subsequently, SVD was performed on the randomised data matrix and the fraction of variation of the inferred singular values compared to the fractions of variation of the unpermuted data (Figure 2). Using multiple randomisations we verified that the null distribution of singular values was very tight (Figure 2), as expected since null singular values reflect a global property of the randomised data, which should be robust to further randomisations of the data. Thus, significant components of variation were selected as those whose variation was larger than the expected variation under the null-hypothesis. This gave five principal components in addition to the top component, making a total of six. The biological significance of the five components of variation (top principal component did not correlate with any phenotype) was tested by correlation to phenotypes of interest and experimental factors (Figure 3). For continuous and ordinal variables (e.g. BSCE, age) associations were evaluated using linear regression, for categorical variables (e.g. Beadchip) we used a Kruskal-Wallis test. For the time to onset of nephropathy (CC-case/control) we used a Cox-regression with time to event = time to onset of nephropathy (NP), and event = 1 (NP), 0 (no NP).

#### Supervised analysis

Association between CpG-valued methylation profiles and diabetic nephropathy was performed using a Cox-regression model where time to event equals the time between diagnosis of T1D to the onset of nephropathy. Multivariate regressions were performed for each CpG separately and included factors for chip, BSCE, sex, cohort and age at sample draw. To correct for multiple testing we estimated the false discovery rate (FDR) using the q-value framework [39]. We have shown previously that the analytical q-value estimates are similar to those obtained using permutations of sample labels preserving the potential correlative structure between CpGs [31].

## Results

### Methylation profile of arrays

**Figure 4:**
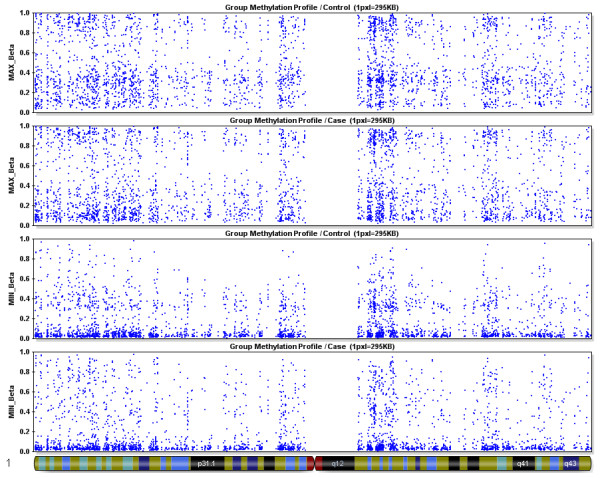
**Maximum and minimum β values for Chromosome 1**. Maximum and minimum β values across the 2903 probed CpGs located on Chromosome 1 for Controls and Cases.

The methylation values as defined by β scores ranging between 1 and 0, or fully to non-methylated, are displayed for all 27,578 CpGs queried as histograms in Figures 1a and 1b, for controls and cases respectively. Both show a similar pattern with a high peak of hypomethylated loci and a low peak in the hypermethylated loci. This is the expected U shape of methylation pattern of the genome but due to the array design which is skewed towards promoter region CpGs in CGIs shows the reverse pattern with a larger peak at the low end of the distribution. The minimum and maximum β values results for a representative chromosome, chromosome 1, are shown in Figure 4. This displays the individual results for the 2,903 probed CpGs that reside across this chromosome, for cases and controls separately. Scatter plots showed highly equivalent intensity scores (r^2 ^= 0.9990) and average β scores (r^2 ^= 0.9996) between cases and controls (data not shown).

### DNA methylation analysis

**Figure 5:**
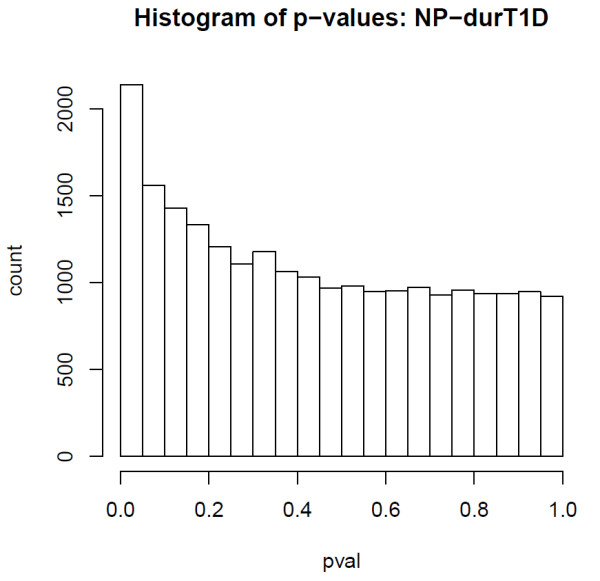
**Nephropathy and duration type 1 diabetes *p *values**. Histogram of p-values after explicit correction for all potentially confounding factors reveals an excess of significant p values for CpGs correlated with Nephropathy and duration of Type 1 Diabetes.

**Figure 6:**
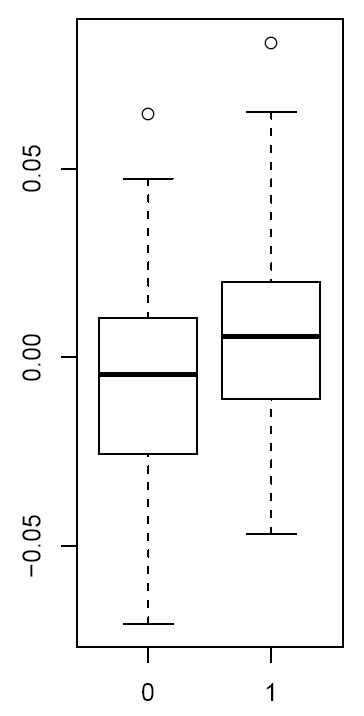
**Z score values for cg07341907**. Box plot for adjusted comparison of methylation Z-score values for the *UNC13B *promoter CpG:cg07341907 on controls (0) and cases (1).

SVD of the normalised data revealed significant components of variation that correlated with age, time to onset of nephropathy, sex, but also with experimental factors including chip, cohort and bisulphite conversion efficiency (Figures 2 and 3). As age and experimental factors could confound the analysis, we identified CpGs correlating with time to onset of nephropathy by applying a multivariate Cox-regression model to each CpG site, including the confounding factors as covariates. We observed that the resulting p-value distribution was significantly different from a uniform distribution with an excess of p-values close to 0, suggesting that a significant number of CpGs are correlated with time to onset of nephropathy (Figure 5). This involves the contribution of directional methylation increases or decreases observed in cases, with respect to the period of time that nephropathy took to develop, and controls, whom had had a variable period of T1D without developing nephropathy. At an FDR = 0.15, we observed 263 CpGs as conferring increased risk and 162 CpGs conferring decreased risk for nephropathy, respectively (Additional File 2: Supplementary Table S1). However, whether these changes are related to cause or effect of early/late onset of nephropathy cannot be answered by this study. Gene set enrichment analysis searches for genes that share common biological function, chromosomal location, or regulation, and this analysis run on these data using the Molecular Signatures Database (MSigDB v2.5) [40] did not reveal any significant associations, after correction for multiple testing. Using a more stringent FDR of 0.05 resulted in a set of 19 CpGs and these are listed in Table 2. The CpG cg07341907, located 5' of the *UNC13B *gene, was the only CpG near a previously identified T1D nephropathy-associated candidate gene within this group. The box plot for adjusted methylation Z-score differences for this CpG is shown in Figure 6, with higher levels of methylation in the case group (0.00557) versus controls (-0.00563) (Uncorrected average β values were 0.147 and 0.157, for controls and cases respectively). The putative association SNP rs13293564 resides within the first intron of this gene [13]. MUNC13, the protein coded for by *UNC13B*, has been shown to be up-regulated in the renal cortex of rats with streptozotocin-induced diabetes and its expression is induced by hyperglycaemia [41].

**Table 2:** Methylation Associations

Ilmn CpG ID	Gene	Gene Function	Location	coef	exp(coef)	se(coef)	z	Pr(> |z|)	Q value
cg01172656	*NRBF2*	Transcription regulation	10:64563126	0.631	1.880	0.136	4.653	3.266 × 10-06	0.031

cg18346038	*PIGU*	GPI-anchor biosynthesis	20:32728489	0.624	1.867	0.136	4.603	4.164 × 10-06	0.031

cg00117172	*RUNX3*	Transcription regulation	1:25128425	0.682	1.977	0.150	4.548	5.427 × 10-06	0.031

cg02068676	*COBRA1*	Negative regulation of transcription	9:139269304	0.662	1.938	0.149	4.447	8.703 × 10-06	0.037

cg00025138	*MAP3K9*	Mixed-lineage kinase	14:70345670	0.543	1.721	0.125	4.335	1.457 × 10-05	0.046

cg03221776	*MFSD3*	Transmembrane transport	8:145704779	0.520	1.682	0.121	4.308	1.644 × 10-05	0.046

cg12181621	*HIST1H3I*	Nucleosome assembly	6:27948047	0.558	1.748	0.132	4.216	2.486 × 10-05	0.048

cg03752885	*DAPK3*	Induction of apoptosis	19:3920736	0.492	1.636	0.118	4.189	2.798 × 10-05	0.048

cg12688215	*KTI12*	ATP binding	1:52271816	0.446	1.563	0.107	4.175	2.976 × 10-05	0.048

cg07341907	*UNC13B*	Exocytosis	9:35151971	0.606	1.833	0.145	4.169	3.056 × 10-05	0.048

cg26850145	*ZBTB5*	Transcription regulation	9:37455023	-0.485	0.616	0.117	-4.142	3.441 × 10-05	0.048

cg21312090	*TRPS1*	NLS-bearing substrate import into nucleus	8:116750901	0.441	1.555	0.107	4.135	3.543 × 10-05	0.048

cg23266266	*DCUN1D4*	-	4:52403466	0.560	1.751	0.136	4.117	3.847 × 10-05	0.048

cg04466870	*SFXN4*	Transmembrane transport	10:120915170	0.477	1.611	0.116	4.102	4.092 × 10-05	0.048

cg16898420	*PPAPR3*	Catalytic activity	9:102830899	-0.711	0.491	0.175	-4.066	4.786 × 10-05	0.048

cg17260725	*CCNB2*	Cell division	15:57184580	0.523	1.687	0.129	4.0596	4.915 × 10-05	0.048

cg17428423	*DOC2A*	Regulation of calcium ion-dependent exocytosis	16:29929633	-0.728	0.483	0.180	-4.053	5.049 × 10-05	0.048

cg18731014	*ZNF639*	Transcription regulation	3:180524284	0.556	1.744	0.137	4.050	5.122 × 10-05	0.048

cg21830413	*VPS26*	Protein transport	10:70554257	0.472	1.604	0.117	4.036	5.430 × 10-05	0.048

Cox-Regression analysis results of CpGs, with FDR (q value) < 0.05, for time to develop nephropathy. (Location given as Chromosome:Base Pair (Build Hs 36.1))

To investigate whether there was a significant co-directional change of CpGs within 1 kb of each other, the normalised results for each of those proximal pairs within this distance were combined and compared in a case versus control analysis, with two of the genes in Table 2, *PPAPR3 *(cg02590345 and cg16898420 - separated by 119 bp) and *TRPS1 *(cg12569516 and cg21312090 - separated by 534 bp) also being in the top ten results of this analysis (data not shown).

## Discussion

This study investigated the methylation state of approximately 14,000 CGIs in promoter regions of genes spread throughout the genome in a cohort of 192 type 1 diabetic patients, half of whom had developed diabetic nephropathy. A Cox-regression model was utilised whereby the effect of a genetic susceptibility has a multiplicative effect over time on the risk of a subject developing nephropathy. This analysis, after accounting for confounding effects, identified an excess of CpG sites which were observed to have a change in methylation status that could be correlated with duration of diabetes prior to onset of nephropathy with a FDR of 0.15. Of these observations 263 CpGs were identified as conferring increased risk and 162 CpGs conferring decreased risk for nephropathy, respectively and co-localised to 421 unique genic regions. However this dataset did not reveal any significant enrichment of particular physiological gene set pathways.

Using a more stringent FDR cut-off of 0.05, a set of 19 CpGs was identified (in 19 unique gene CGIs). One CpG in this list, cg07341907 is located 18 bp upstream of the transcription start site of a previously identified type 1 diabetic nephropathy gene, *UNC13B *[13]. Levels of methylation for this CpG were shown to be slightly higher in the case group compared with controls. Whilst expression of this gene has been shown to be increased in models of nephropathy and hyperglycaemia [41] the expression effect of methylation changes can often be difficult to predict [42]. The other CpG assayed within this genic region, cg02096633, is located ~0.5 kb further downstream, 197 bp into the first intron and shows consistently low methylation levels in both cases and controls (β-value averages 0.051 and 0.052 respectively). The susceptibility SNP, rs13293564 resides within the first intron and in Caucasians (from CEU HapMap data) is within a linkage disequilibrium block of 23 kb that includes the CGI. Tregouet *et al*. could determine no plausible function for the intronic SNP, however did identify five SNPs in strong linkage disequilibrium with this SNP that reside within the plausible promoter region [13]. Two of which (rs10081672 and rs10972333) were found to affect potential transcription factor binding sites. Additionally, the former SNP (rs10081672) creates or abrogates a CpG site depending on which allele is present. Therefore, modification of the promoter region of this gene whether genetically or epigenetically or in combination could influence its expression by affecting transcription factor binding. Tregouet *et al*. proposed that *UNC13B *mediates apoptosis in glomerular cells due to hyperglycemia, and they therefore suggested that this association could indicate initiation of nephropathy [13].

Whilst the 27K Infinium assay enables high-throughput investigation of individual CpG sites with high resolution, and is a considerable improvement on the previous Golden-Gate methylation assay, this methodology still has some limitations. Only approximately 0.1% of the total number of CpGs within the genome are assessed, however the placement of these CpGs are predominately within the CpG Islands with high likelihood of critical effects on promoter activity. As these CpGs can significantly affect or silence expression, changes can be more deleterious than gene sequence variants, therefore increasing the potential power of the assay. Additionally not all CpG sites need to be assessed as correlation of CpG status between sites can extend up to 1 kb [43]. Whist dramatic switching on or off facilitated by DNA methylation may occur within these promoter regions, more subtle and perhaps dynamic effects may however be missed that are occurring in the surrounding CGI shores (regions within 2 kb of the islands) [44]. Changes in gene body methylation that may influence the expression of certain isoforms, by effecting the inclusion or exclusion of certain exons, are also not assayed. Additionally this array does have a bias towards cancer and imprinted genes; with ~200 of these having 3-20 CpG probes per promoter. Increasing knowledge with regards to the most biologically informative CpG sites and future up scaling will undoubtedly improve targeted platforms, although a more in depth investigation of all of the CpGs of the genome would require a differing approach. An enrichment technique such as MeDIP-Seq would gain a representative genome wide picture but this lacks the base pair resolution of this array [45]. Isolated studies utilising full bisulphite sequencing of a very small number of genomes have recently been published [26], however scaling up to large numbers of cases and controls currently remains prohibitive due to cost.

Like all genome-wide association studies this study was designed to find statistically significant associations, in this case with DNA methylation variation not with genotype, but not to identify the underlying mechanism(s) for the cause or function of the observed variation. Although DNA methylation is tissue-specific, examination of peripheral whole blood was informative in this case in order to determine whether any genome-wide methylation signal changes could be detected in this easily accessible surrogate tissue. Whilst only a small number of significant sites were identified, due to the stringent False Discovery Rate cut-off implemented, this experiment did identify possible biomarkers in this pathogenic process with more easily implementable clinical utility potential. Therefore, this indicates that active disease processes can be identified in the DNA methylation pattern of peripheral blood and be a possible marker of these [31]. The development, as well as the progression, of diabetic nephropathy has been linked to the same inflammatory cell changes that are implicated in the progression of T1D itself, such as the activated T cells involved in the destruction of islet β cells [7]. Furthermore, additional inflammatory-related or other signals may be present in peripheral blood DNA that require isolation of specific cell types to detect and other methylation change signals may only be able to be identified in the affected renal cells themselves.

## Conclusion

This study is one of the first to investigate a complex disease trait utilising this high-throughput DNA methylation 27 K array based assay. The effect of length of T1D status before developing or not developing nephropathy was observed to show a correlation with respect to methylation scores of individual CpG loci. By the examination of a cohort of 192 T1D patients this study has confirmed the utility of this approach to genome wide DNA methylation analysis and the potential prospects for larger and subsequent replication studies of epigenomic factors in common diseases.

## List of Abbreviations

BSCE: Bisulphite Conversion Efficiency; CGI: CpG Island; ESRD: End-Stage Renal Disease; FDR: False Discovery Rate; NP: Nephropathy; T1D: Type 1 Diabetes Mellitus; SVD: Singular Value Decomposition.

## Competing interests

The authors declare that they have no competing interests.

## Authors' contributions

CGB participated in the design of the study, Bisulphite conversion, analysis and drafting of the manuscript. AET performed the statistical analysis. VRK participated in the design of the study and coordination. DAS, APM and SB conceived of the study, participated in its design and coordination, and drafting of the manuscript. All authors read and approved the final manuscript.

## Pre-publication history

The pre-publication history for this paper can be accessed here:

http://www.biomedcentral.com/1755-8794/3/33/prepub

## Supplementary Material

Additional file 1 **Supplementary Figure S1**. Distribution of CpG sites on Infinium platform. (Top, Left) Chromosome Distribution of CpG sites. (Top, Right) Distance to Transcription Start Site of CpG Locus. (Bottom, Left) Interrogated CpG Islands: near genes, near miRNA. (Bottom, Right) Number of CpG sites per CpG Island. Click here for file

Additional file 2 **Supplementary Table S1**. List of CpGs with q value < 0.15. Column headings are IlmnID = Illumina CpG ID, Gene_ID = Entrez Gene ID, Symbol = Gene Symbol, coef = coefficient, exp(coef) = exponential of coefficient, se(coef) = standard error of coefficient, z = z score, Pr(> |z|) = probability greater than absolute z score, q value Click here for file
