# Supplementary figures and images for: Genome-wide DNA methylation analysis for diabetic nephropathy in type 1 diabetes mellitus
Source: BMC Med Genomics. 2010 Aug 5;3:33. doi: 10.1186/1755-8794-3-33 (PMC2924253; doi:10.1186/1755-8794-3-33)

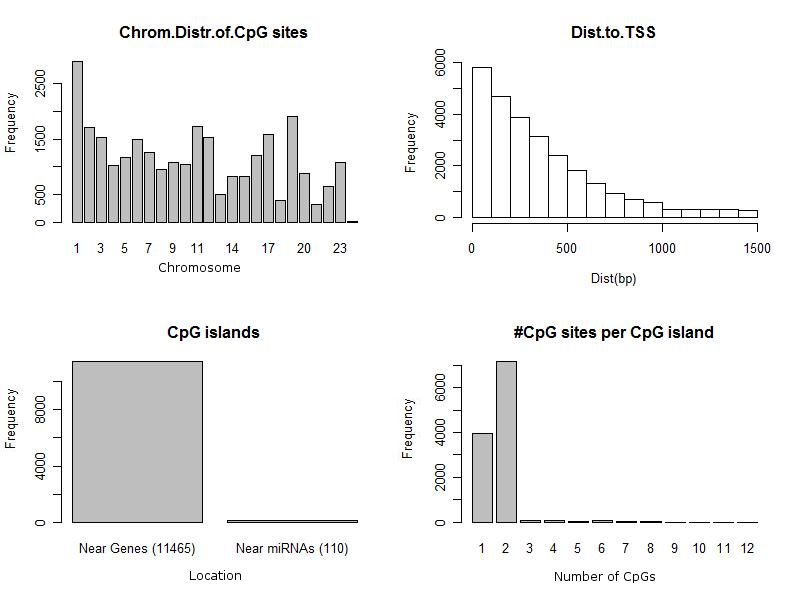

Supplement: Additional file 1 — Supplementary Figure S1. Distribution of CpG sites on Infinium platform. (Top, Left) Chromosome Distribution of CpG sites. (Top, Right) Distance to Transcription Start Site of CpG Locus. (Bottom, Left) Interrogated CpG Islands: near genes, near miRNA. (Bottom, Right) Number of CpG sites per CpG Island. [file 1755-8794-3-33-S1.PNG]
